# Contaminants from a former Croatian coal sludge dictate the structure of microbiota in the estuarine (Raša Bay) sediment and soil

**DOI:** 10.3389/fmicb.2023.1126612

**Published:** 2023-02-09

**Authors:** Weiting Zhang, Qianyun Mo, Zaixing Huang, Muhammad Adnan Sabar, Gordana Medunić, Tatjana Ivošević, Huan He, Michael Urynowicz, Fang-Jing Liu, Hongguang Guo, Rizwan Haider, Muhammad Ishtiaq Ali, Asif Jamal

**Affiliations:** ^1^Key Laboratory of Coal Processing and Efficient Utilization of Ministry of Education, School of Chemical Engineering and Technology, China University of Mining and Technology, Xuzhou, China; ^2^Department of Civil and Architectural Engineering, University of Wyoming, Laramie, WY, United States; ^3^Environmental Risk Control Engineering Laboratory, Division of Environmental Design, Kanazawa University, Kanazawa, Japan; ^4^Department of Geology, Faculty of Science, University of Zagreb, Zagreb, Croatia; ^5^Faculty of Maritime Studies, University of Rijeka, Rijeka, Croatia; ^6^College of Safety and Emergency Management and Engineering, Taiyuan University of Technology, Taiyuan, China; ^7^Institute of Energy & Environmental Engineering, University of the Punjab, Lahore, Pakistan; ^8^Department of Microbiology, Quaid-i-Azam University, Islamabad, Pakistan

**Keywords:** Raša coal, microbial diversity, estuary, PAHs, hazardous trace elements, natural attenuation

## Abstract

**Introduction:**

Croatian superhigh-organic-sulfur Raša coal had been mined for nearly 400 years. The release of hazardous trace elements (HTEs) and toxic organic pollutants (TOPs) into the local environment by coal mining, preparation, and combustion activities has resulted in pollution.

**Methods:**

In this study, the diversity and composition of microbial communities in estuarine sediment and soil samples as well as community function responses to the pollutants were investigated.

**Results:**

The results showed that PAH degradation does occur following 60 years of natural attenuation, the location is still heavily polluted by polycyclic aromatic hydrocarbons (PAHs) and HTEs. Microbial analyses have shown that high concentrations of PAHs have reduced the diversity and abundance of microbial communities. The pollution exerted an adverse, long-term impact on the microbial community structure and function in the brackish aquatic ecosystem. Microorganisms associated with the degradation of PAHs and sulfur-containing compounds have been enriched although the diversity and abundance of the microbial community have reduced. Fungi which are believed to be the main PAH degrader may play an important role initially, but the activity remains lower thereafter. It is the high concentrations of coal-derived PAHs, rather than HTEs, that have reduced the diversity and abundance of microbial communities and shaped the structure of the local microbiota.

**Discussion:**

This study could provide a basis for the monitoring and restoration of ecosystems impacted by coal mining activities considering the expected decommission of a large number of coal plants on a global scale in the coming years due to growing global climate change concerns.

## 1. Introduction

Coal is the second largest fuel accounting for ∼30% of the world’s total primary energy, with a consumption of 150 EJ in 2020 (BP, 2021). However, coal mining, processing, and combustion have led to the release of hazardous trace elements (HTEs) (e.g., chromium, selenium, molybdenum, vanadium) and toxic organic pollutants (TOPs, e.g., polycyclic aromatic hydrocarbons [PAHs], sulfur heterocycles, nitrogen heterocycles, oxygen heterocycles) into aquatic and terrestrial ecosystems, which may have a long-term impact on the local microflora. Studies have shown that elevated levels of HTEs and TOPs persist in the environment, accumulate in soils, sediments, and organisms, and deteriorate many inland and coastal aquatic environments; many are also potential human mutagens (Addink and Olie, 1995; Johnson, 1995; Nadal et al., 2004; Medunić et al., 2018, 2021; Yu et al., 2019; Fei et al., 2020; Zhang J. et al., 2020; Li, 2021; Li et al., 2021).

The reduction of microbial diversity in the local environments, due to the release of TOPs and HTEs from coal mining, processing, and combustion processes, has been well documented (Ma et al., 2016; Zhao et al., 2019; Hamidović et al., 2020). In addition, microbes capable of degrading PAHs [e.g., *Pseudomonas putida* (Ghiorse et al., 1995; Herrick et al., 1997), *Alphaproteobacteria*, *Betaproteobacteria*, *Cupriavidus*, *Luteimonas* (Ma et al., 2016)] and resistant/tolerant to HTEs [e.g., Proteobacteria, Firmicutes (Huang et al., 2019; Roth et al., 2019; Zhao et al., 2019), Acidobacteria, Bacteroidetes (Roth et al., 2019; Zhao et al., 2020)] have been found in coal-contaminated soils and sediments. While the effects of HTEs and TOPs on microorganisms in coal-contaminated environments have received considerable attention, the ecological consequences of the complex combined pollution associated with coal wastes on microbial assemblages in natural settings, especially aquatic environments, remain largely unknown.

**FIGURE 1:**
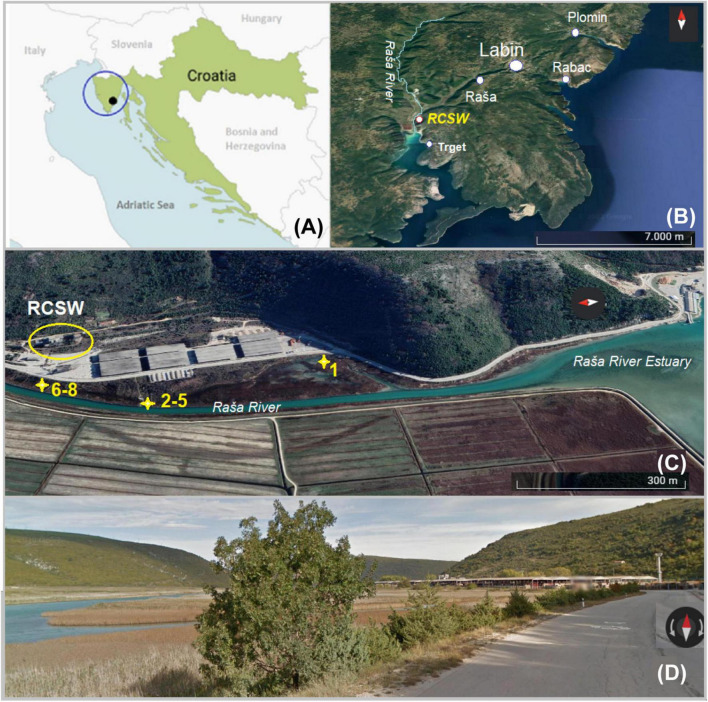
Map of the study area: **(A)** Position of the study area (black dot) on Istrian Peninsula (blue circle) in Croatia (Europe); **(B)** position of sampling sites on the eastern part of Istria with Raša River and position of coal processing facility (RCSW); **(C)** position of sampling sites No. 1-8; **(D)** street view of the sampling area.

Croatian superhigh-organic-sulfur (SHOS) Raša coal has a very long mining history of nearly 400 years (Medunić et al., 2019b). A Raša coal separation and washing facility (RCSW), situated within Raša Bay (Figure 1), was in operation till the 1960s. Although mining of Raša coal ceased on November 29, 1999, due to economic and construction difficulties, a total of about 40 Mt of coal has been excavated, while an estimate of more than 4 Mt of Raša coal remains underground (Medunić et al., 2020a). Studies have shown that Raša coal has SHOS content of up to 9-11%. Previous publications characterized the detrimental impacts of Raša coal on the local environment regarding elevated levels of HTEs (Se, U, Mo, and V in particular) and PAHs in local soil, crops, groundwater, surface water, and sediments (Medunić et al., 2016, 2020a, 2020b, 2019a, 2019b, 2021). However, the extent of the impact of the RCSW on downstream estuarine ecosystems including Raša Bay has remained unknown. In an effort to investigate these effects, soil and sediment samples were collected from the site where Raša coal sludge (wastewater from coal preparation) was disposed of directly into Raša Bay between the 1930s and 1960s, and thereafter remained abandoned. Downstream sediment samples were also collected for comparison. The objectives of this study were as follows: 1) to examine the composition of collected soil and sediment samples in terms of HTEs and PAHs; 2) to explore the long-term effects of the coal-derived contaminants on the structure and function of the microbial community of the local estuarine environment. The site provides a unique opportunity to get an insight into a microbial community structure in a karst aquatic ecosystem that has been affected by coal-derived pollutants following long-term (>60 years) natural attenuation processes.

## 2. Materials and methods

### 2.1. Study site and sampling

The study site is an estuarine karst environment that can be characterized by wetland features. It is located inside Raša Bay (Figure 1) belonging to the north part of the Adriatic Sea, in a Štalije settlement, coordinates of 45°02′50′′N and 14°02′82′′E. The area is characterized by hard carbonate bedrock overlain by reddish clay-loam cambic type of soil. The karst topography and residue of variable thickness from the weathering of carbonate strata typify the terrain. The study area has a Mediterranean type of climate, with mild humid winters (annual precipitation of 900–1000 mm), hot dry summers, and prevailing NE winds (the so-called bora) (Durn et al., 1999; Peh et al., 2010). Since the local area is composed mainly of highly vulnerable karst, its environmental quality should be carefully monitored (Medunić et al., 2020a).

The sampling campaign was carried out on October 5, 2021. Sediment and soil samples were collected from sites (No. 1–8) within some 500–700 m from the RCSW as indicated in Figure 1. Samples No. 1-7 were collected from surface soil and sediment (0-5 cm). The position of samples No. 6–8 corresponds to the site of former lagoons (a naturally shallow part of the estuary) that received coal sludge (coal processing wastewater) some 60-90 years ago. There, huge quantities of wastewater were stored and gradually filled up with coal and mud particles. Today, it is partly covered by grass and shrub vegetation. Samples No. 2–5 were collected downstream from the lagoon along Raša River. Sample No. 1 was collected from the estuary sediment located halfway between the local road and the coastline, partly wetland. Three samples with different depths were collected at site No. 8 with a stainless soil core sampler that was partly filled with groundwater. The samples were designated as No. 8-10, 8-20 and 8-30, corresponding to the depths of 15–20, 20–30, and 30–40 cm. Following the return to the lab, the samples were divided into two portions. One subset was stored at −80 °C for microbial DNA extraction. The other subset was air-dried, and then carefully packed in sterile bags for further analyses.

### 2.2. Analytical methods

#### 2.2.1. Characterizations of soil and sediment samples

Water content, total organic carbon (TOC, dissolved), total nitrogen (TN), total sulfur (TS), total organic sulfur (TOS), and pH of the samples were determined. Briefly, the samples were dried at 105°C to determine the moisture. Any animal and plant residues, and stones were removed after drying. The samples were ground and passed through a 2-mm sieve. Then, the samples were mixed with DI water at a sample-to-water ratio of 1:5 (w/v) with continuous mixing for 4 h, and filtered with membrane filters (0.45 μm, F513132, Sangon Biotech Co. Ltd) prior to TOC and TN analyses (TOC-L TNM-L CSN, Shimadzu, Japan) (Jones and Willett, 2006; Li et al., 2017). TS and TOS were measured using an automatic sulfur meter (CTS3000, Xuzhou Terui Instrument and Equipment Co., Ltd., Xuzhou, China) (Lange and Brumsack, 1977; Bohn et al., 2002). Sample pH was determined with a soil-to-water ratio of 1:2.5 (w/v) using a pH meter (PHS-25, Shanghai INESA Scientific Instrument Co., Ltd., Shanghai, China) (Liang et al., 2017).

#### 2.2.2. Heavy metal analysis

Heavy metals including Se, Mo, Pb, U, V, Cr, Cu, Zn, Sr, and Cd were determined using an inductively coupled plasma mass spectrometry (ICP-MS). An aliquot of 0.5 g dry samples was digested in a TeFlon-coated vessel with 10 mL of digestion solution. The digestion solution was prepared by mixing 2 volumes of hydrofluoric acid, 1 volume of hydrogen peroxide, 1 volume of nitric acid, and 1 volume of perchloric acid. Then, the samples were sequentially heated in a microwave oven under the following conditions: 150°C for 30 min, 180°C for 60 min, 190°C for 60 min, and 210°C for 30 min. After heating, the samples were allowed to cool down before filtering. The filtrate was transferred to a 100 mL volumetric flask with 5.0% v/v HNO_3_ as the makeup solution. Then the concentrations of these metals were determined using an ICP-MS (ICP-RQ, Thermo Scientific, USA). Certified standards of these elements were purchased from Solarbio Science and Technology Co., Ltd (Beijing, China) and used for calibration. Rhodium served as an internal standard (Medunić et al., 2021).

#### 2.2.3. Organic pollutants analysis

PAHs were determined with the S-PAHGMS01 method based on the US EPA 8270 and ISO 18287 with a GC/MS. The GC/MS (8890/5977B, Agilent, USA) was equipped with an HP-MS column (30 m × 0.25 μm × 0.25 mm, Agilent, USA). 10 g dried samples were extracted with 50 mL dichloromethane three times with ultrasonication. The GC/MS was run as follows: injection volume, 1 μL; injection inlet temperature, 300 °C; running mode, split mode with a split ratio of 10:1. Helium was used as the carrier gas with a constant flow rate of 1.0 mL/min. The initial temperature of the column was 40 °C and held for 4 min. Then the column was heated to 300 °C at a rate of 10 °C/min and held at 300 °C for 2 min. The MS was operated in an electron ionization (EI) mode at 70 eV. The temperature of the ion source was set at 230 °C. The fragmental ions were scanned in the quadrupole analyzer at 150 °C at a range of m/z 30–500. The solvent delay was set at 3 min. Total ion chromatograms (TICs) and mass spectra were processed using MSD Productivity ChemStation software (Agilent, USA). NIST11 library was used for compound identification (Gworek et al., 2018).

#### 2.2.4. DNA extraction and sequencing

The DNA samples were extracted with an E.Z.N.A.^®^ soil DNA kit (Omega Bio-Tek, Norcross, GA, USA) according to the manufacturer’s instructions. The bacterial and archaeal 16S rRNA genes and fungal ITS1 genes were respectively amplified with the primer pairs 338F (ACTCCTACGGGAGGCAGCAG)/806R (GGAC TACHVGGGTWTCTA-AT) (Claesson et al., 2009), 524F10extF (T GYCAGCCGCCGCGGTA-A)/Arch958RmodR (YCCGGC-GTT-G AVTCCAATT) (Zhang J. et al., 2020), and ITS1F (CTTGGTCAT-TTAGAG-GAAGTAA)/ITS2R (GCTGCGTTCTTCATCG-ATGC) (Orgiazzi et al., 2012) using a therm-ocycler PCR system (GeneAmp 9700, ABI, U.S). Then the purified PCR amplicons were pooled in equimolar and paired-end sequenced (2 300) on an Illumina Miseq platform (Illumina, San Diego, USA) at Majio Bioinformatics Technology Co., Ltd. (Shang hai, China). Operational taxonomic units (OTUs) were clustered with a 97% similarity cutoff using the UPARSE pipeline (version 7.1). Sequences of OTUs were classified within the Sliva database release 138 for bacteria and archaea, and Unite INSD release 8.0 (Unite and International Nucleotide and Sequence Databases) for fungi. The data were analyzed on the Majorbio I-Sanger Cloud Platform (www.i-sanger.com). The sequencing data for our study are available in the NCBI Sequence Read Archive under accession no. PRJNA880742.

#### 2.2.5. Predicted metagenomic analysis

Marker gene data and reference genomes can be used to predict genes’ functions in a genome. As a computational tool, PICRUSt 2 (phylogenetic investigation of communities by reconstruction of unobserved states) was used to predict the functional potential of the microbial community. In this study, the sediment microbial assemblages exhibited a relatively low mean NSTI (Nearest Sequenced Taxon Index) value (0.18+−0.06), implying that the prediction was reliable for subsequent functional analysis of the microbial community. The predicted genes were then used to compare with the Kyoto Encyclopedia of Genes and Genomes (KEGG) pathways. However, it does have limitations for fungi, as it only provides information on enzymes (Langille et al., 2013; Douglas et al., 2020).

#### 2.2.6. Statistical analysis

Bivariate correlation (2-tailed) analyses including Pearson or Spearman coefficients were performed for the correlations between microbial diversities and environmental factors, as well as between different environmental factors of soil and sediment samples. To explain the correlation between fungal guilds and Naphthalene, PAHs and sulfur genes, Person and bivariate correlation (2-tailed) analyses were used. Levene’s test was applied to test the equality of variances. Subsequently, the mean difference was checked by the appropriate t-test method. All statistical analyses were performed on SPSS 26.0 with a P-value less than 0.05 considered statistically significant.

## 3. Results

### 3.1. Physicochemical characteristics and levels of PAHs and HTEs in samples

The physiochemical properties of the collected samples and their PAH concentrations are shown in Table 1. The samples had generally alkaline pH (7.6-8.3). Depending on the collection sites, moisture ranged widely from 19% to 77%. Total organic carbon ranging from 98 to 268 mg/kg and C:N ratio between 4.8 and 26.0 were recorded in the samples. Elevated levels of TS (up to 9.3%) and OS (up to 4.2%) were detected in samples No. 6-8 (Figure 1), which represent the former coal sludge-receiving site. Interestingly, the pH, moisture, C:N ratio, TS and OS of sampling sites No. 1-5 were significantly different from No. 6-8 (ANOVA, P < 0.05).

**TABLE 1:** Physiochemical characteristics, concentrations of toxic organic pollutants and hazardous trace elements of samples.

Sample ID	1	2	3	4	5	6	7	8-20	8-30	8-40	a	b	c	Unit
pH	7.59	7.69	7.70	7.87	7.84	8.21	8.22	8.21	8.28	8.23	/	/		/
Moisture	77.22	32.80	30.40	30.80	34.00	23.25	15.11	18.87	19.52	22.47	/	/		%
TOC	123.2	106.0	146.7	268.7	106.8	97.7	123.5	153.1	132.6	120.2	/	/		mg/kg
C/N	17.3	11.1	14.6	5.0	4.8	25.9	22.6	14.6	20.3	24.3	/	/		/
TS	1.2	1.3	1.4	1.3	1.5	9.3	4.8	4.6	3.8	3.0	/	/		%
OS	0.2	0.2	0.3	0.2	0.4	3.7	4.2	3.4	2.9	2.3	/	/		%
Naphthalenes	0.0	0.0	0.0	0.0	0.0	35.8	40.1	33.6	31.3	26.3	/	0.03		mg/kg
Dibenzothio-phene	0.0	0.0	0.0	0.0	0.0	8.9	7.6	7.9	7.5	7.4	/	/		mg/kg
PAHs	0.0	0.0	0.0	0.0	0.0	44.8	47.7	41.5	38.7	33.7	/	0.1		mg/kg
Se	19.9	21.9	32.7	22.9	22	38.5	34.1	32.8	26.9	28.2	0.3 2.3	1.3	/	mg/kg
Mo	58.3	55.5	27.7	16.4	12	20.5	8.9	12.1	9.4	8.5	/ /	2.4	/	mg/kg
Pb	28.2	26.4	21.3	18.6	19.7	12.3	19.7	15.9	15.4	19.3	17 26	37.1	48	mg/kg
U	6.9	3.7	3.4	2.9	2.9	42.9	33.9	20	9.9	8.4	2.7 5.0	4.4	/	mg/kg
V	72.9	86.2	73.1	75.9	73.6	199.3	86.3	139.7	105.2	121.6	90 98	203.5	148	mg/kg
Cr	119.9	217.7	181.8	167.1	188.6	115.9	114.7	137.3	143.3	170.7	80 64	181.8	60	mg/kg
Cu	80.8	40.6	36.3	37.7	36.2	18.7	31	42.6	30.7	37	25 19	31.9	31	mg/kg
Zn	210.5	80.7	67.2	100.4	93	23.6	42.3	49.8	39.7	59.7	70 80	126.2	108	mg/kg
Sr	247.2	333.9	300	264.7	262.8	303.5	261	306.1	292.3	322.4	240 187	99.2	117	mg/kg
Cd	0.2	0.2	0.2	0.2	0.2	0.2	0.2	0.3	0.3	0.2	0.3 1.8	0.9	0.4	mg/kg

TOC, total organic carbon (dissolved); C/N, C:N ratio; TS, total sulfur; OS, organic sulfur; Naphthalenes, including Naphthalene, 2-methyl-, Naphthalene, 1-methyl-, Naphthalene, 1, 5-dimethyl-, Naphthalene, 1, 3-dimethyl-, Naphthalene, 2, 7-dimethyl- and Naphthalene, 2, 3-dimethyl-. a: soil and sediment (<2 mm, total world median data); b: uncontaminated topsoil in Rabin as a reference; c: element values representative of the coastal Croatian background. Underlined values refer to sediment reference levels (Medunić et al., 2016).

Two types of PAHs including naphthalene and its derivatives [naphthalenes], and dibenzothiophene [DBT]) were quantified (Table 1). PAHs and heavy metals in an uncontaminated soil sample in the same area from our previous studies were served as references (Medunić et al., 2016). Results revealed that the samples contained extremely high concentrations of naphthalene (26.3-40.1 mg/kg) and DBT (7.4-8.9 μg/kg) from the coal sludge-receiving site compared to downstream locations (0 μg/kg) and control sample (0.1 μg/kg). The correlations of physicochemical properties with PAHs (Supplementary Table 1) were not significantly affected by the depth at site No. 8 except for TS and OS. In addition, for samples from all sites, correlation analysis revealed that C:N ratio and PAHs (Spearman ρ = 0.692, *P* = 0.027), TS and PAHs (Spearman ρ = 0.925, *P* = 0.000), OS and PAHs (Spearman ρ = 0.937, *P* = 0.000) were positively correlated (Supplementary Table 2). Although the concentrations of specific PAH congeners varied from site to site, the anomalously high levels of PAHs in No. 6-8 samples indicate that the site is still heavily polluted from the former coal sludge disposal.

Table 1 also shows that the concentrations of major HTEs in the samples are considerably higher compared with the references (Medunić et al., 2016). The concentrations of selenium, uranium and vanadium in samples No. 6-8 are significantly higher than that in samples No. 1-5, while lead and zinc showed the opposite (ANOVA, ρ < 0.05). Furthermore, positive correlations of Se-U-V (Spearman ρ > 0.69,*P* < 0.05) and Pb-Zn-Cu (Spearman ρ > 0.71,*P* < 0.05) were observed which might be worthy of further investigation.

### 3.2. Diversity and abundance of microbial communities

A total of 610,122, 682,950, and 788,679 high-quality bacterial, archaeal, and fungal sequences were obtained and grouped into 8,231, 607 and 4,722 OTUs using a 97% sequence similarity cutoff. Then OTU tables were, respectively, rarified to the minimum depth of sequences of 30,709, 43,999, and 52,799 per sample.

**FIGURE 2:**
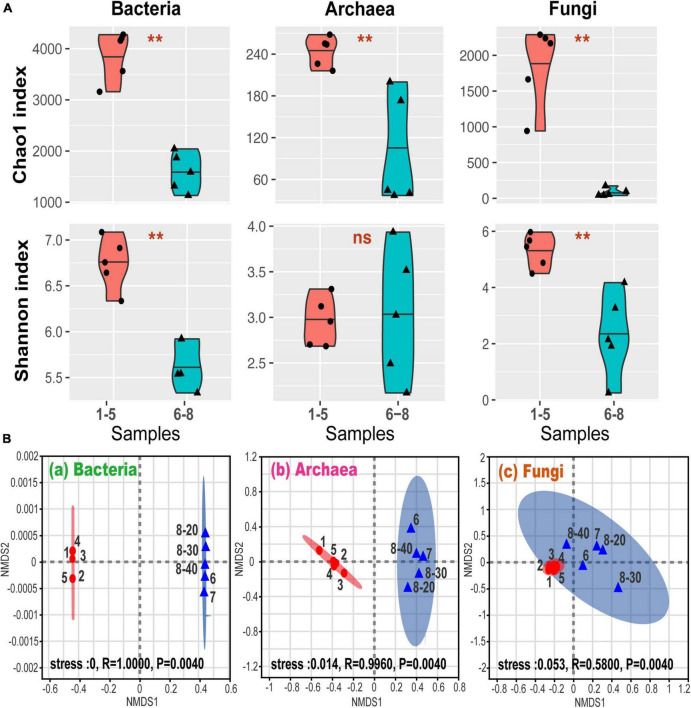
**(A)** Alpha-diversity via Wilcoxon rank sum test. ^**^P < 0.01; ns, *P* > 0.05; **(B)** general patterns of microbial beta-diversity. NMDS showed the structure of microbial community for sample bacteria (a), archaea (b), and fungi (c). 95% confidence ellipses were shown around the samples. Similarity values among the samples between ex-coal separation unit and other place were examined via the ANOSIM test, which are shown in each plot.

Figure 2A shows alpha-diversity levels of bacteria, archaea, and fungi which were all higher in samples No. 1-5 than in samples No. 6-8, except for the Shannon index for the archaea. The community diversity and abundance of bacteria, archaea and fungi were not significantly affected by the depth, suggesting that bacterial, archaeal and fungal communities were not sensitive to the soil and sediment depth at site No. 8. These were confirmed by the correlation analysis (Supplementary Table 1). Furthermore, the differences in the alpha-diversity indices between the coal sludge-receiving site and the downstream locations were confirmed via the Wilcoxon rank sum test (Figure 2A). It appears that except for archaeal diversity, the microbial diversities and abundance were significantly higher in the downstream locations than in the contaminated site.

Non-metric multidimensional scaling (NMDS) analysis revealed that samples No. 1-5 and No. 6-8 formed distinct clusters (Figure 2B), with significant differences being found at OTU levels (analysis of similarity test [ANOSIM]). These differences among samples No. 1-5 and No. 6-8 were the highest for bacterial communities, followed by archaeal and fungal communities, indicating that the coal wastewater discharge impacted the bacterial communities the most.

### 3.3. Microbial community analyses

**FIGURE 3:**
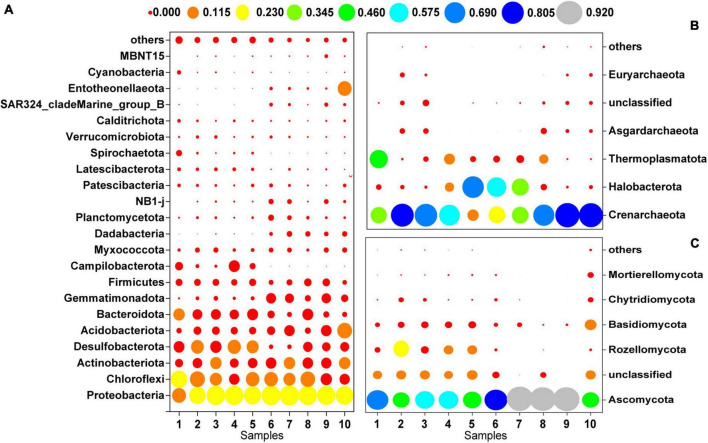
Microbial community composition at the phylum level (>0.1%); **(A)** bacteria, **(B)** archaea, **(C)** fungi.

Figure 3 shows the microbial community composition at the phylum level for a relative abundance greater than 0.1%. Bacterial sequences were primarily composed of the phyla Proteobacteria (27.92%), Chloroflexi (15.47%), Actinobacteriota (9.59%), Desulfobacterota (9.15%), Acidobacteriota (7.25%), and Bacteroidota (7.12%). The majority of the archaeal sequences belonged to the phyla Crenarchaeota (54.19%), Halobacterota (21.27%), and Thermoplasmatota (11.48%). The most abundant fungal phyla were Ascomycota (72.06%), Rozellomycota (7.45%) and Basidiomycota (6.98%).

**FIGURE 4:**
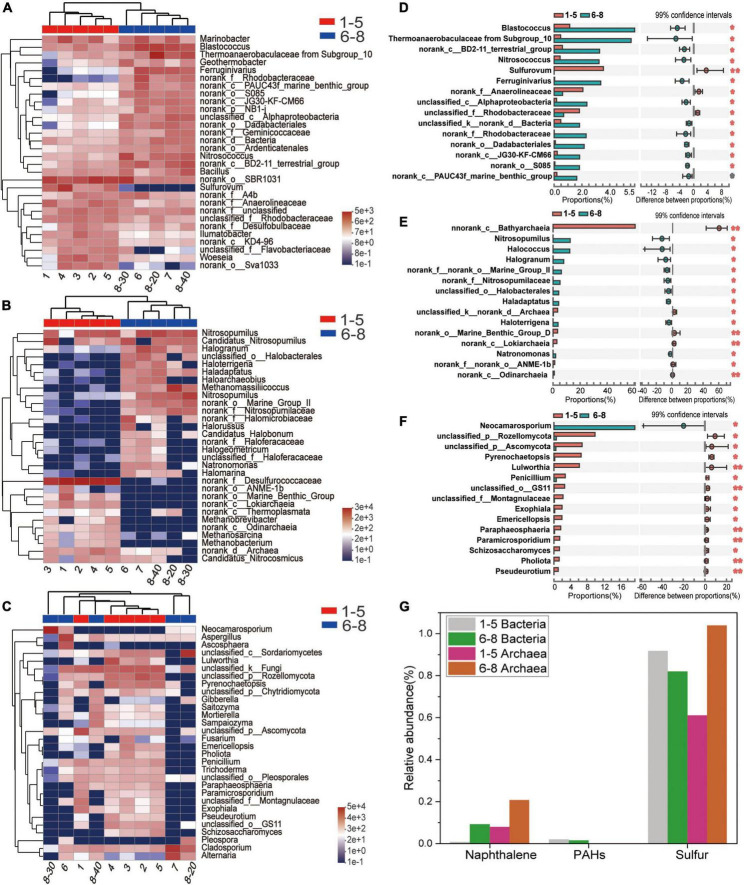
**(A–C)** Heatmap diagrams of the dominant 30 genera; **(A)** bacteria, **(B)** archaea, **(C)** fungi; **(D–F)** comparisons of **(D)** bacterial, **(E)** archaeal and **(F)** fungal abundances with significant differences at the genus level. **(G)** Relative abundance (%) of PAHs-, Naphthalene- and sulfur-degradation genes inferred by PICRUSt 2 in the communities from coal-waste contaminated site.

Microbial community profiles at the genus level were demonstrated by a hierarchically clustered heatmap (Figures 4A–C). The top 30 genera and 10 samples were both hierarchically clustered based on the Bray-Curtis similarity index. Bray-Curtis dissimilarity analysis revealed that microbial communities of the 10 samples were clustered into two groups. The bacterial and archaeal clustering indicated that samples 1-5 (the downstream locations) were closely related while samples 6-8 (the coal sludge-receiving site) shared a close relationship. Conversely, samples No. 6, 8-30 cm, and 8-40 cm exhibited a distant relationship with samples No. 7 and 8-20 cm in fungal communities. This affinity relationship was also intuitively demonstrated by the NMDS analysis. As shown in Figures A–C, the most representative bacterial and archaeal genera in samples No. 6-8 were *Blastococcus* and *Halogranum*, respectively. In samples No. 1-5, *Sulfurovum*, *Sulfurimonas*, norank_f_Desulfurococcaceae, *Methanosarcina*, and *Methanobacterium* were the most abundant genera. Although most of the dominant genera of samples No. 1-5 and No. 6-8 were different, the top 30 genera were shared by all the samples. This may represent the ecological coherence of the water and sediment of Raša river.

Wilcoxon Rank Sum tests were conducted (double tail test, fdr multiple correction) to determine the difference in abundance of the classified bacterial, archaeal and fungal taxa between the sample groups of No. 1-5 and No. 6-8. The species with significant differences in the sum of the means in the top 15 genera are presented in Figures 4D–F. Interestingly, most of these bacteria and archaea were significantly enriched in samples No. 6-8. Specifically, *Blatococcus*, “Subgroup 10” from Thermoanaerobaculaceae, *Nitrosococcus*, *Ferruginivarius*, *Nitrosopumilus*, *Methanomassiliicoccus*, *Halogranum*, Halobacterales (order), *Haladaptatus*, *Haloterrigena*, and *Natronomonas* were enriched in samples No. 6-8 (all *P* < 0.05). The same members in samples 1-5 were all less than 0.1%. Conversely, most fungi were significantly enriched in samples No. 1-5, while only one genus showed an abundant advantage in samples No. 6-8, i.e., *Neocamarosporium*. In comparison, members of *Sulfurovum*, Anaerolineaceae (family), Rhodobacteraceae (family) and Bathyarchaeia (class) were enriched in samples No. 1-5. The members belonging to *Sulfurovum* and Bathyarchaeia (class) accounted for 3.54% and 60.6% of the population in samples No. 1-5 but less than 0.01% samples No. 6-8.

### 3.4. Impacts of sample properties on the microbial community structure and function

**FIGURE 5:**
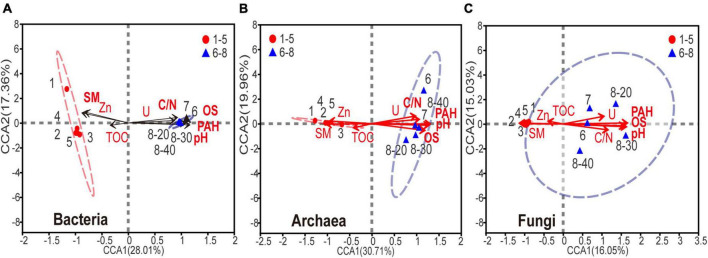
Canonical correspondence analysis of **(A)** bacterial, **(B)** archaeal, **(C)** fungal community composition and the subset of seven environmental variables (including SM, PAHs, DBT, OS, TS, Se, V, C/N and pH). The environmental variable in bold font represents *P* < 0.05.

Canonical correspondence analysis (CCA) was used to determine the relationship between biochemical factors and bacterial, archaeal or fungal communities. Because the strong multicollinearity was detected among nine HTEs (variance inflation factor (VIF) value > 10), VIF analysis was performed prior to the CCA analysis (threshold value = 2). As shown in Supplementary Table 3, two HTEs’ environmental factors, Zn and U, were ruled out. As shown in Figure 5, for the eight environmental variables, acute angles were formed among variables of PAHs, U, C:N ratio (C/N), pH, and OS, indicating that these variables had a synergetic impact on the microbial community. On the contrary, sample moisture and Zn showed an opposite relationship in obtuse angles on the microbial composition with the other five variables (PAHs, U, C/N, pH, OS). Apparently, the microbial composition of samples No. 6-8 is positively correlated with the levels of PAHs, U, C/N, pH, and OS, and negatively associated with the sample moisture, thus implying that a higher level of PAHs, U, C/N, pH, and OS, and lower moisture might reduce the abundance and diversity of microbial communities in soils and sediments. All the edaphic variables explained 92.3, 93.4, and 88.1% of the bacterial, archaeal, and fungal variance, respectively. The key environmental factors impacting microbial communities in the samples were pH, OS, and PAHs (*P* < 0.05).

Provided that the microbial diversity and community composition are different between sample groups No. 1-5 and No. 6-8, the key functional microorganisms between the two groups were compared and presented in Figure 4G. Notably, 21 genes (including pcaG, pcaH, ligA, ligB, phdF), 23 genes (including adh, frmA, ADH5, nahF), and 16 genes (including dmsB, dmsC, ssuE, msuE, and cysJ) were affiliated with important enzyme categories (dehydrogenase, hydroxylase, aldolase, decarboxylase, hydrolase) involved in the degradation of PAHs (Supplementary Figure 1), naphthalene (Supplementary Figure 2), and sulfur-containing compounds (Supplementary Figure 3). These pathways were predicted to be harbored by the soil and sediment bacteria and archaea. Among these genes, the sulfur degradation-related genes generally showed the highest abundance followed by naphthalene and PAHs (Figure 4G). The abundance of functional genes associated with naphthalene and sulfur degradation in samples No. 6-8 was greater than that in samples No. 1-5. Notably, most of the genera with significant differences in the top 15 bacterial and archaeal genera have functional genes that are related to the degradation of naphthalene, PAHs, or sulfur-containing compounds. Specifically, the genus, *Ferruginivarius* can degrade all three substances while the other 5 genera (*Natronomona*, *Blastococcus*, *Halogranum*, *Haladaptatus*, *Haloterrigena*) could degrade two substances (Naphthalene/PAHs, sulfur-containing compounds). Both groups exhibited a large number of genes that are involved in sulfur metabolism. In addition, total enzymes and enzymes capable of PAHs degradation for fungi in the two sample groups have shown almost no difference (Table 2), suggesting that indigenous fungi contribute little to the degradation of PAHs in the long run.

**TABLE 2:** Enzyme abundance of fungi by PICRUSt 2.

Enzyme description	1.10.3.2 laccase	1.11.1.7 peroxidase	Total
1	258,963	69,508	81,588,426
2	175,760	39,306	82,295,204
3	187,184	42,986	79,146,167
4	218,509	46,861	81,964,137
5	201,701	43,532	74,664,812
6	211,914	51,321	75,415,107
7	397,246	99,963	73,362,268
8-20	256,325	63,343	77,798,089
8-30	307,892	102,323	71,780,019
8-40	208,579	32,854	75,643,813

## 4. Discussion

### 4.1. High concentrations of PAHs and HTEs in the contaminated samples

Elevated concentrations of HTEs were documented in all collected samples (Table 1), thus indicating that the Raša Bay area in the vicinity of the RCSW is severely polluted with HTEs as a result of the legacy of coal sludge. Previous publications report that the local environment (e.g., groundwater, soil, wild plants, crops, and aquatic sediment) is polluted with HTEs due to former coal mining, processing, and combustion activities (Sando et al., 2015; Medunić et al., 2016, 2020a,2020b,^2021^). Interestingly, the concentrations of Mo, Cu, Zn, Cr, and Pb in samples No. 1-5, especially Zn and Pb, were higher than that in samples No. 6-8, suggesting migration occurred possibly from the erosion and upstream leaching of the coal sludge-receiving site (No. 6-8), while Se, U, and V are coalphilic elements (Yudovich and Ketris, 2006). However, Mo, Cu, Zn, Cr, and Pb are less coalphilic, and more soluble in water (Medunić et al., 2016). Therefore, leaching of Mo, Cu, Zn, Cr, and Pb from coal debris contained in the sludge-receiving site soil could have happened. A previous study (Medunić et al., 2016) also indicated positive correlation (*P* < 0.05) between Cu-Pb-Zn and Se-U-V clusters in soil polluted by Raša coal.

PAHs are components of fossil fuels such as coal. DBT and its derivatives are the main sulfur-containing aromatic compounds in fuels (Kertesz and Wietek, 2001). These aromatic compounds and their degradation products have been found in groundwater, seawater, sediment, soil and atmospheric samples related to old gas plants, industrial waste sites and wood treatment facilities (Warshawsky, 1992; He et al., 2009), which can persist in the environment (Xu et al., 2006; He et al., 2009). According to the classification proposed by Maliszewska-Kordybach (1996), the former coal sludge-receiving site (samples No. 6-8) is heavily polluted (>> 1,000 μg/kg, Table 1). On the contrary, PAHs were not detected in the downstream samples (samples No. 1-5). Notably, the PAH composition of coal is commonly dominated by an average of 4.5-ring PAHs (Dong et al., 2012), while those detected in the analyzed samples were dominated by a lower number of rings (2-rings). In addition, concentrations of PAHs were also lower compared with raw Raša coal (53,407.7 μg/kg) (Medunić et al., 2016). These results indicate that PAHs were not migration-prone and were naturally attenuated by the soil and sediment microbes (Schneider et al., 1996; Coates et al., 1997; Yagi et al., 2010). However, the concentration of naphthalene from the polluted coal sludge-receiving site was apparently higher than coal-tar waste (Yagi et al., 2010). This is likely due to slow desorption rates for PAH compounds in coal-contaminated floodplain soils (Achten et al., 2011).

Complex variation occurred in sample properties due to coal processing wastewater, such as increases in sample pH and C:N ratio. These changes could be partly explained by microorganisms, whose activities have proven essential for the functioning of these nutrient cycles (Falkowski et al., 2008; Dai et al., 2018; Jiao et al., 2018). Studies have shown that when pH was between 8 and 9, the activities of phenanthrene, pyrene and naphthalene-degrading microorganisms were highest (Song et al., 2012; Lian et al., 2015). Furthermore, a suitable C:N ratio is beneficial for the continuous growth and proliferation of PAH-degrading microorganisms in soil and sediment (Cai et al., 2021). However, these environmental changes can also lead to an alteration of microbial communities (Schneider et al., 1996; Chaput et al., 2015; Jiao et al., 2018; Liu et al., 2018), as shown by the canonical correspondence analysis (Figure 5). On the other hand, a high concentration of sulfur was detected in the polluted coal sludge receiving site, attributed to Raša coal which has exceptionally high values of sulfur (up to 9-11%). Noteworthy, previous study also confirmed that sulfur and PAHs were strongly correlated in soil polluted with Raša coal as a consequence of a local coal-fired power plant (Spearman, *P* < 0.01) (Medunić et al., 2016).

### 4.2. PAHs reducing the diversity and abundance of microbial community in the polluted site

HTEs have been repeatedly illustrated to have significant impacts on microbial community structure and function across diverse habitat types (Nicomrat et al., 2006; Weber et al., 2008; Khan et al., 2010; Liu et al., 2018; Zhao et al., 2021). Although this study showed that the levels of HTEs were elevated in all samples, CCA analysis revealed that HTEs may not have played an important role in the change of microbial communities (Figure 5, all *P* > 0.05). On the contrary, the accumulation of PAHs has been the main cause for changing the structure of microbial communities and thus their functions. Since HTEs cannot be degraded like TOPs, they may not participate in the basic element cycle of cells (Nies, 1999). Furthermore, it has been reported that microbial cells have ubiquitously defensive mechanisms against HTEs in such environments (Nies, 2003). In this study, there are bacteria and archaea with intrinsic high-level resistance to HTEs that have been reported in other studies [e.g., *Bacillus* sp. (Amoozegar et al., 2005), *Marinobacter* sp., *Halococcus* sp. (Voica et al., 2016)].

This study shows that PAHs decreased the biodiversity of sample microbiomes, which is in accordance with the fact that high concentrations of PAHs negatively affect the total microbial populations (Zoppini et al., 2016; Picariello et al., 2020). PAHs have detrimental impacts on microbial metabolic processes, enzyme activities (Picariello et al., 2020), and cell functional integrity (Zoppini et al., 2016). For example, PAHs can inhibit oxidative phosphorylation processes by preventing the ATP-forming mechanism (Zoppini et al., 2016). This would explain why the PAH pollution of the Raša coal sludge-receiving site is one of the key factors affecting the microbial community at this study locality. Studies showed that fungal biomass and enzyme activities associated with PAH degradation (e.g., laccase, peroxidase, cytochrome P450 monooxygenase) increased in the early PAH contamination (Marco-Urrea et al., 2015; Mineki et al., 2015; Jové et al., 2016; Bellino et al., 2019; Picariello et al., 2020). However, a minuscule difference in enzyme activity related to PAH degradation was observed in this study. This suggests that fungi have few roles to play in the later stage of the degradation of PAHs, while bacterial communities were more responsive and adaptive to contamination than fungi. This could be ascribed to the high intrinsic growth rates and resilience in disturbances and perturbations of bacteria, rendering a more rapid response to environmental changes such as coal processing wastewater pollution in this study. This contrasting pattern could reflect that bacteria and archaea were more sensitive to PAH pollution from the coal sludge-receiving site (No. 6-8).

### 4.3. Enrichment of genera capable of degrading organic pollutants

Recent research has revealed significant influences of coal waste-associated pollution on the composition and diversity of indigenous soil microbial communities (Ghiorse et al., 1995; Yagi et al., 2010; Roth et al., 2019; Hamidović et al., 2020). This study shows that Proteobacteria, Chloroflexi (bacteria), Crenarchaeota, Halobacterota (archaea), Ascomycota, and Basidiomycota (fungi) were the dominant phyla in the coal sludge-receiving site (affected by the RCSW). Similar phyla have previously been found to dominate the soil microbiota in coal-related wastes (Yagi et al., 2010).

Noteworthy, while the top 30 genera were shared by all samples, a very different distribution pattern of dominant genera was observed. Specifically, *Blastococcus*, “Subgroup 10” from Thermoanaerobaculaceae, *Nitrosococcus*, Ferruginivarius (bacteria), *Nitrosopumilus*, *Methanomassiliicoccus*, *Halodaptatus*, Halobacterales (order) (archaea), and *Neocamarosporium* (fungi) were significantly more abundant in the polluted coal sludge samples (*P* < 0.01). Interestingly, *Blastococcus*, *Ferruginivarius*, “Subgroup 10” from Thermoanaerobaculaceae, *Methanomassiliicoccus*, *Halodaptatus*, and Halobacterales (order) have shown the ability to catabolize complex aromatic compounds or simple hydrocarbons (Joo and Kim, 2005; Youssef et al., 2012; Dyksma et al., 2016; Zárate et al., 2021; Vidal-Verdú et al., 2022). Additionally, “Subgroup 10” from Thermoanaerobaculaceae (Basen et al., 2018), *Nitrosococcus* (Dyksma et al., 2016), *Ferruginivarius* (Wiese et al., 2020), *Halodaptatus* and Halobacterales (order) (Wang et al., 2022) are involved in the sulfur oxidation process. “Subgroup 10” from Thermoanaerobaculaceae has strong viability in polluted coal sludge soil and may have participated at the end of the sulfur cycle in the degradation of TOPs and benefit the growth of other microorganisms (Basen et al., 2018). Besides, *Nitrosococcus* can release nitrogen and nitrogen oxides (Watson, 1965), which explains why samples No. 6-8 have a higher C:N ratio. Subsequent functional inference of communities further revealed that *Blastococcus*, *Ferruginivarius*, “Subgroup 10” from Thermoanaerobaculaceae, *Methanomassiliicoccus*, *Halodaptatus*, and Halobacterales (order) may have relevant genes involved in the PAH and naphthalene degradation. “Subgroup 10” from Thermoanaerobaculaceae, *Nitrosococcus*, *Ferruginivarius*, *Halodaptatus* and Halobacterales (descending order) may have relevant genes involved in the sulfur-metabolism pathways. Overall, our results demonstrate that the native community in the study area contains microbes that can tolerate high concentrations of HTEs and degrade PAHs and sulfur-containing organic compounds. Notably, the microorganisms that can degrade refractory and sulfur-containing organics may have been enriched as they adapt to the surrounding environment. However, the restoration (biodegradation process) of the polluted ecosystem is slow as extremely high levels of PAHs and HTEs are still observed today, following some 60 years of natural attenuation processes. This study shows that polluting organic compounds mostly shape the microbial structures in the local estuarine environment.

## 5. Conclusion

In the present study, we have quantified PAHs and HTEs in estuarine sediments and soils polluted by coal sludge that has been abandoned for over 60 years. The effects of the two classes of contaminations on microbial diversity and community structure have been investigated. The results show that PAH degradation does occur by the natural attenuation as indicated by the lower number of mean PAH rings and lower concentrations of PAHs compared with raw Raša coal. However, the site is still heavily polluted by extremely high concentrations of PAHs, while fingerprint elements (Se, U, Mo, and V) of superhigh-organic-sulfur Raša coal were also elevated in the analyzed samples. Microbial analyses revealed that the community structure of the polluted coal sludge-receiving site was significantly different from the downstream. Gemmatimonadota, Dadabacteria and Halobacterota at the phylum level and *Blastococcus*, “Subgroup 10” from Thermoanaerobaculaceae, *Nitrosococcus*, *Ferruginivarius*, *Ntrosopumilus*, *Methanomassiliicoccus*, *Natronomonas, Halogranum*, *Haladaptatus* and *Haloterrigena* at the genus level were the dominant microorganisms in the polluted samples. Further analysis of microbial functionalities has shown that microorganisms associated with the degradation of PAHs and sulfur-containing compounds have been enriched although the diversity and abundance of the microbial community have reduced. Fungi which are believed to be the main PAH degrader may play an important role initially, but the activity remains lower thereafter. It is the high concentrations of coal-derived PAHs, rather than HTEs, that have reduced the diversity and abundance of microbial communities and shaped the structure of the local microbiota. Furthermore, this study also revealed that the restoration of sites polluted by persistent chemicals like PAHs and HTEs by natural attenuation processes is not sufficiently effective. Further anthropogenic intervention may be needed to expedite the remediation process. This study also contributes to the ecological risk assessment of coal-related pollution and bioremediation of such environments. Although the analyses are limited by one-time sampling, a well-planned long-term monitoring program is strongly recommended.

## Data availability statement

The data presented in this study are deposited in the NCBI Sequence Read Archive repository, accession number: PRJNA880742.

## Author contributions

WZ: data curation, formal analysis, investigation, methodology, visualization, conceptualization, and writing—original draft/reviewing and editing. HH: formal analysis, resources, and writing—original draft/reviewing and editing. QM: data curation, methodology, and writing—original draft/reviewing and editing. MS: data curation, formal analysis, and visualization. GM: data curation, formal analysis, investigation, methodology, visualization, and writing—reviewing and editing. TI: data curation and visualization. MU: formal analysis and writing—original draft/reviewing and editing. F-JL: resources and methodology. HG, RH, MA, and AJ: writing—original draft/reviewing and editing. ZH: formal analysis, conceptualization, supervision, resources, and writing—original draft/reviewing and editing. All authors contributed to the article and approved the submitted version.
