# Corrigendum: Contaminants from a former Croatian coal sludge dictate the structure of microbiota in the estuarine (Raša Bay) sediment and soil

**DOI:** 10.3389/fmicb.2023.1175748

**Published:** 2023-03-28

**Authors:** Weiting Zhang, Qianyun Mo, Zaixing Huang, Muhammad Adnan Sabar, Gordana Medunić, Tatjana Ivošević, Huan He, Michael Urynowicz, Fang-Jing Liu, Hongguang Guo, Rizwan Haider, Muhammad Ishtiaq Ali, Asif Jamal

**Affiliations:** ^1^Key Laboratory of Coal Processing and Efficient Utilization of Ministry of Education, School of Chemical Engineering and Technology, China University of Mining and Technology, Xuzhou, China; ^2^Department of Civil and Architectural Engineering, University of Wyoming, Laramie, WY, United States; ^3^Environmental Risk Control Engineering Laboratory, Division of Environmental Design, Kanazawa University, Kanazawa, Japan; ^4^Department of Geology, Faculty of Science, University of Zagreb, Zagreb, Croatia; ^5^Faculty of Maritime Studies, University of Rijeka, Rijeka, Croatia; ^6^College of Safety and Emergency Management and Engineering, Taiyuan University of Technology, Taiyuan, China; ^7^Institute of Energy & Environmental Engineering, University of the Punjab, Lahore, Pakistan; ^8^Department of Microbiology, Quaid-i-Azam University, Islamabad, Pakistan

**Keywords:** Raša coal, microbial diversity, estuary, PAHs, hazardous trace elements, natural attenuation

In the published article, there was an error in the **Funding** statement. The grant number “2019XKQYMS57” for Fundamental Research Funds for the Central Universities of China was incorrect. The correct Funding statement appears below.

