# Supplementary material for: Contaminants from a former Croatian coal sludge dictate the structure of microbiota in the estuarine (Raša Bay) sediment and soil
Source: Front Microbiol. 2023 Feb 9;14:1126612. doi: 10.3389/fmicb.2023.1126612 (PMC9947854; doi:10.3389/fmicb.2023.1126612)
Supplement: Supplementary file 1 [file Data_Sheet_1.docx]

**Supplementary Materials**

**Supplementary tables**

Supplementary Table 1. Correlation analysis between physicochemical properties and soil depth, PAHs and soil depth, hazardous trace elements and soil depth, microbial diversity indices and soil depth at site No. 8.

| Variable 1 | Tests of Normality | | | Variable2 | Pearson | | Spearman | |
| --- | --- | --- | --- | --- | --- | --- | --- | --- |
|  | Statistics | df | Sig. |  | Correlation  coeﬃcient | Sig. | Correlation  coeﬃcient | Sig. |
| Depth | 1.000 | 3 | 1.000 | / | / | / | / | / |
| pH | 0.942 | 3 | 0.537 | Depth | 0.277 | 0.821 | / | / |
| Moisture | 0.880 | 3 | 0.325 | Depth | 0.938 | 0.225 | / | / |
| TOC | 0.980 | 3 | 0.730 | Depth | -0.990 | 0.090 | / | / |
| C/N | 0.990 | 3 | 0.807 | Depth | 0.995 | 0.064 | / | / |
| TS | 1.000 | 3 | 1.000 | Depth | -1.000^**^ | 0.000 | / | / |
| OS | 0.997 | 3 | 0.900 | Depth | -0.999^*^ | 0.033 | / | / |
| PAHs | 0.974 | 3 | 0.692 | Depth | -0.987 | 0.103 | / | / |
| HTEs | 0.885 | 3 | 0.341 | Depth | 0.177 | 0.886 | / | / |
| Bacteria-Chao | 0.888 | 3 | 0.350 | Depth | 0.943 | 0.217 | / | / |
| Bacteria-Shannon | 0.970 | 3 | 0.669 | Depth | 0.642 | 0.556 | / | / |
| Archaea-Chao | 0.768 | 3 | 0.041 | Depth | / | / | 0.500 | 0.667 |
| Archaea-Shannon | 0.916 | 3 | 0.439 | Depth | 0.957 | 0.187 | / | / |
| Fungi-Chao | 0.890 | 3 | 0.354 | Depth | 0.759 | 0.451 | / | / |
| Fungi-Shannon | 0.992 | 3 | 0.833 | Depth | 0.574 | 0.611 | / | / |

TS, total sulfur; OS, organic sulfur; C/N, C:N ratio; TOC, total organic carbon (dissolved); HTEs, hazardous trace elements; Sig., significant; ** P < 0.01; * P < 0.05.

Supplementary Table 2. Spearman correlation analysis of different environmental factors of soil and sediment samples.

| Variable 1 | Tests of Normality | | | Variable 2 | Spearman | |
| --- | --- | --- | --- | --- | --- | --- |
|  | Statistics | df | Significant |  | Correlation  coeﬃcient | Significant |
| PAHs | 0.745 | 10 | 0.003 | / | / | / |
| C/N | 0.936 | 10 | 0.513 | PAHs | 0.692* | 0.027 |
| TS | 0.784 | 10 | 0.009 | PAHs | 0.925** | 0.000 |
| OS | 0.809 | 10 | 0.019 | PAHs | 0.937** | 0.000 |
| Se | 0.929 | 10 | 0.439 | / | / | / |
| U | 0.770 | 10 | 0.006 | Se | 0.705* | 0.023 |
| V | 0.786 | 10 | 0.010 | Se | 0.685* | 0.029 |
|  |  |  |  | U | 0.754* | 0.012 |
| Pb | 0.946 | 10 | 0.616 | / | / | / |
| Zn | 0.801 | 10 | 0.015 | Pb | 0.705* | 0.023 |
| Cu | 0.751 | 10 | 0.004 | Pb | 0.707* | 0.022 |
|  |  |  |  | Zn | 0.709* | 0.022 |

** P < 0.01; * P < 0.05.

Supplementary Table 3. Variance inflation factor analysis on Hazardous trace elements (threshold value = 2).

|  | Se | Mo | Pb | U | V | Cr | Cu | Zn | Sr |
| --- | --- | --- | --- | --- | --- | --- | --- | --- | --- |
| VIF before screening | 6.2 | 7.4 | 175.9 | 148.6 | 196.1 | 18.4 | 28.8 | 251.2 | 131.6 |
| VIF after screening | / | / | / | 1.4 | / | / | / | 1.4 | / |

**Supplementary figures**


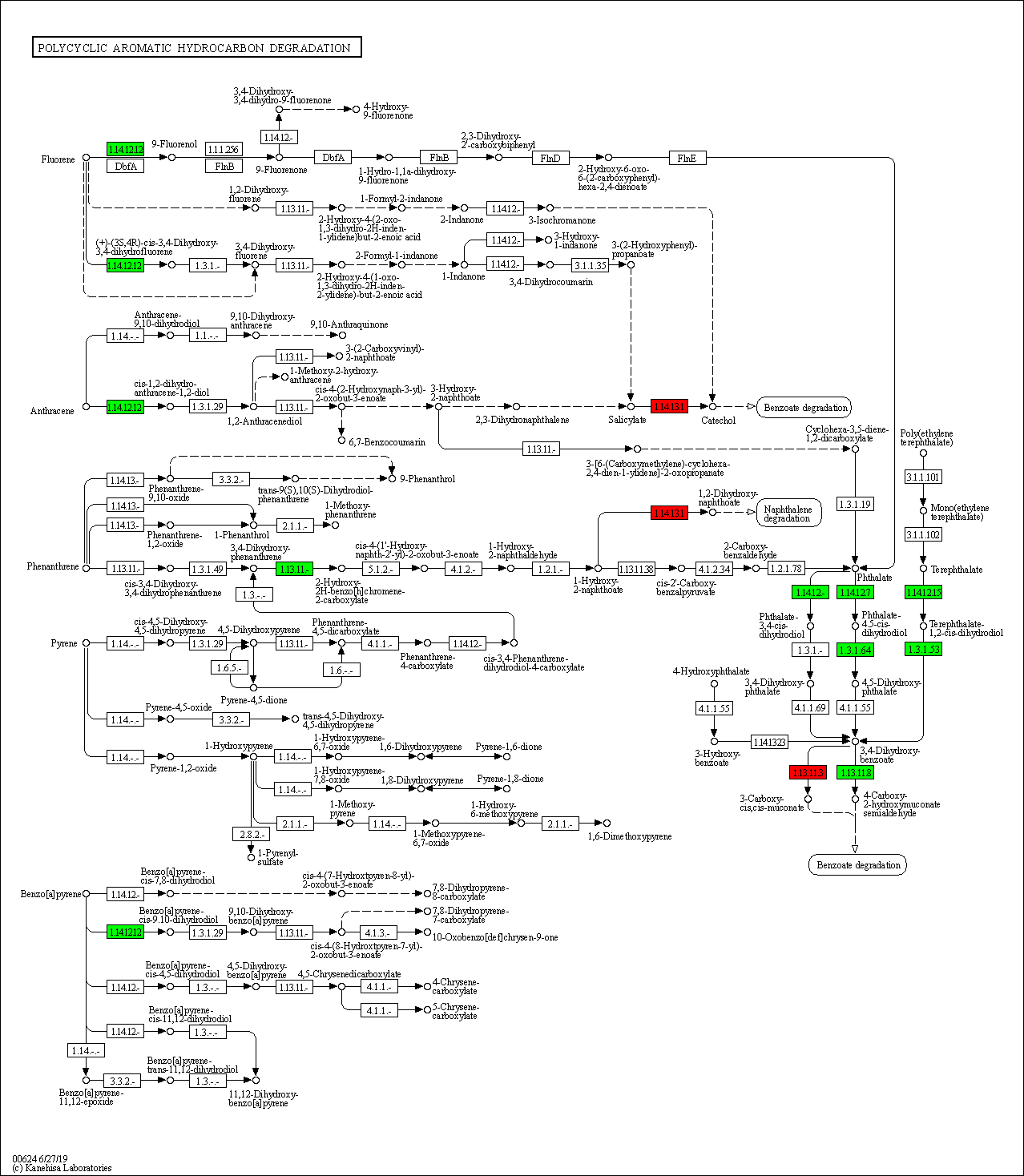


Supplementary Figure 1. Map of PAHs degradation pathway in the KEGG database. The red boxes represent the common functional genes of bacteria and archaea, and the green boxes represents the unique functional genes of bacteria, which were inferred in the sediment communities using PICRUSt 2.


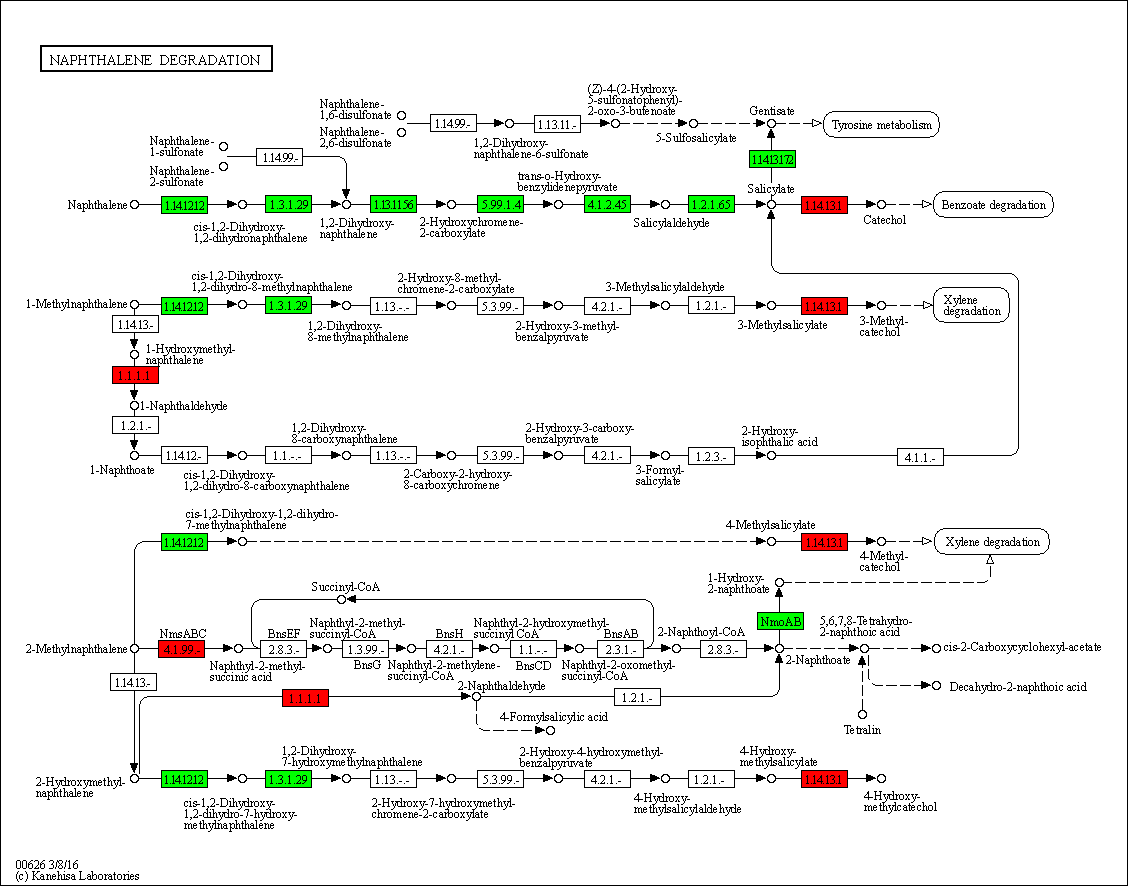


Supplementary Figure 2. Map of Naphthalene degradation pathway in the KEGG database. The red boxes represent the common functional genes of bacteria and archaea, and the green boxes represents the unique functional genes of bacteria, which were inferred in the sediment communities using PICRUSt 2.


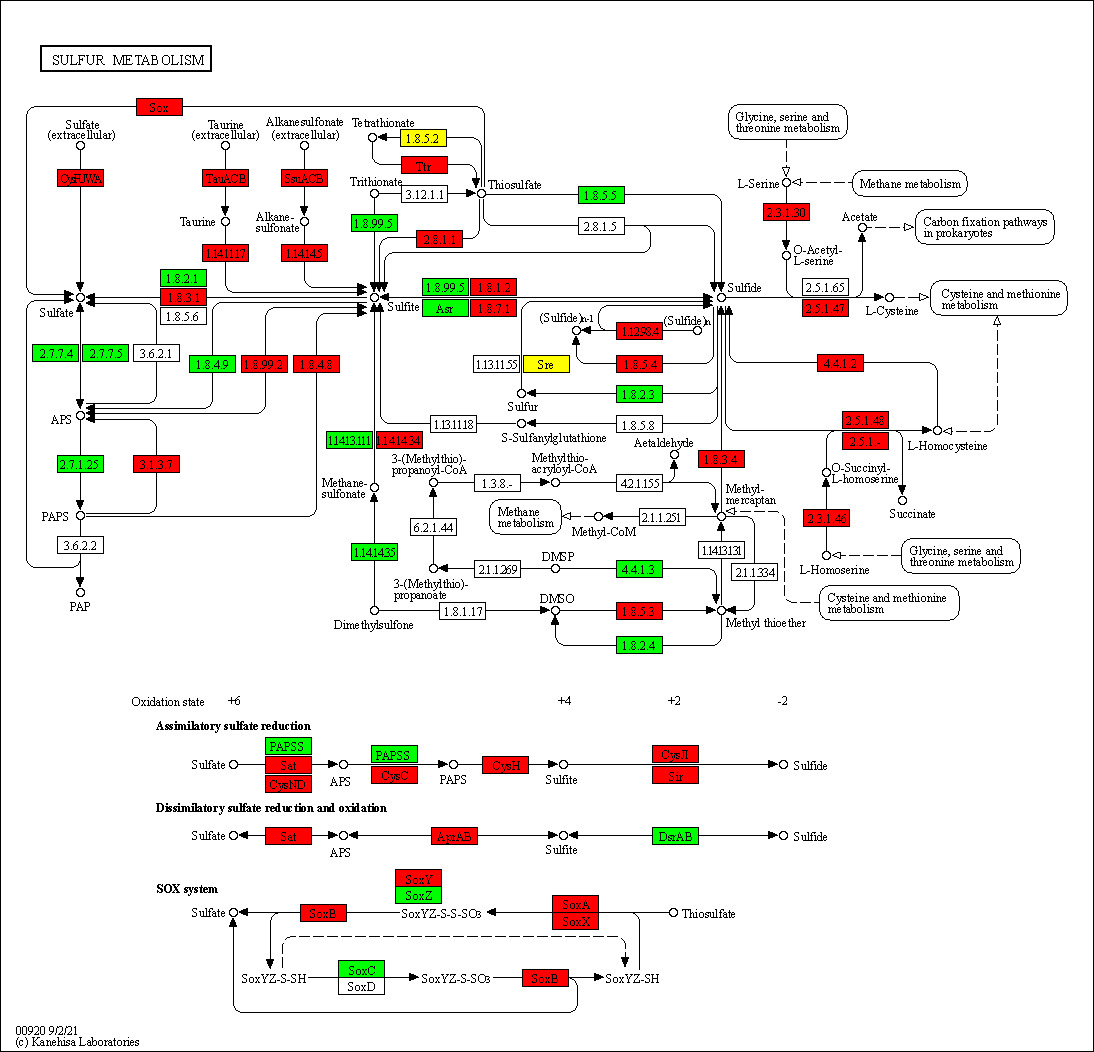


Supplementary Figure 3. Map of Sulfur degradation pathway in the KEGG database. The red boxes represent the common functional genes of bacteria and archaea, the green boxes represent the unique functional genes of bacteria, and the yellow boxes represent the unique functional genes of archaea, which were inferred in the sediment communities using PICRUSt 2.
